# The embryo stage at fresh ET does not affect the cumulative live birth rate in women with a thin endometrium: a retrospective matched-controlled cohort study

**DOI:** 10.3389/fendo.2024.1448138

**Published:** 2024-12-11

**Authors:** Qiao-Song Han, Yan-Hua Chen, Bin Zhang, Jing-Yan Song, Ying Xu, Heng-Bing Li, Zi-Zhen Guo, Zhen-Gao Sun

**Affiliations:** ^1^ The First Clinical College, Shandong University of Traditional Chinese Medicine, Jinan, China; ^2^ Reproductive Medicine Center, Shanxi Maternal and Child Health Care Hospital, Taiyuan, China; ^3^ The Second Affiliated Hospital of Shandong University of Traditional Chinese Medicine, Jinan, China; ^4^ Reproductive Center of Integrated Medicine, The Affiliated Hospital of Shandong University of Traditional Chinese Medicine, Jinan, China

**Keywords:** thin endometrium, fresh ET, blastocyst-stage, cleavage-stage, cumulative live birth rate

## Abstract

**Background:**

The blastocyst-stage embryo has been considered more advantageous for increasing the cumulative live birth rate (CLBR) at fresh embryo transfer (ET) compared to the cleavage-stage embryo. However, it remains uncertain whether this advantage extends to specialized subpopulations, such as women with thin endometrium (TE), who are characteristic of impaired endometrial receptivity. Thus, this study aims to evaluate the difference in the CLBR between cleavage-stage and blastocyst-stage embryos at fresh ET specifically in women with TE.

**Methods:**

A retrospective cohort comprising 1089 women from three centers, ranging from September 2017 to January 2022, was established. These women were diagnosed with TE (defined as endometrium thickness <= 8 mm) and underwent their first fresh ET. To create a comparable cohort between the cleavage and blastocyst groups while adjusting for key covariates, the propensity score matching (PSM) method was employed. The primary outcome assessed was the CLBR per woman. Both cohorts underwent Kaplan-Meier analysis, Cox proportional hazard models, cumulative incidence function (CIF) curve analysis, and Fine-Grey competing risk models to ascertain the impact of embryo stage at fresh ET on CLBR. Additionally, a sensitivity analysis was conducted within a subgroup defining thin endometrium as an endometrium thickness (EMT) < 7 mm.

**Results:**

In the matched cohort after PSM, the CLBR was comparable between groups (p=0.331). However, the cleavage-stage fresh ET was associated with an elevated risk of low birth weight (LBW) (p=0.005) and small for gestational age (SGA) (p=0.037). Kaplan-Meier analysis showed that the median number of embryo transfer cycles was 2 in the cleavage group and 3 in the blastocyst group. The CLBR for the cleavage group reached 78.1%, while the blastocyst group reached 60.0% after 5 cycles of embryo transfers (log-rank test, p=0.09). A multivariable Cox proportional hazard model indicated no significant association between the embryo stage at fresh ET and CLBR (HR=0.80, 95% CI=0.60-1.07). The CIF curve and Fine-Grey competing risk models demonstrated similar results. These analyses were repeated in the original cohort before PSM and in the subgroup with EMT < 7 mm, and the results remained robust.

**Conclusion:**

For TE women receiving fresh ET, the choice between the cleavage-stage embryo and the blastocyst-stage embryo yields comparable CLBR. However, selecting the cleavage-stage embryo is associated with increased risks of LBW and SGA births.

## 1 Introduction

The quality of the embryo and endometrial receptivity are critical determinants of the success of *in vitro* fertilization and embryo transfer (IVF-ET) (1). The trend of extending culture to the blastocyst-stage before embryo transfer (ET) is gaining global acceptance (2). This approach is favored because blastocyst-stage embryos undergo a natural selection process and exhibit improved synchronization with the endometrium, leading to higher clinical pregnancy rates (CPR) and live birth rates (LBR) in fresh ET cycles (3). However, the superiority of blastocyst-stage fresh ET in terms of the cumulative live birth rate (CLBR) remains contentious (4, 5). Some studies have reported higher CLBR with blastocyst-stage fresh ET compared to cleavage-stage fresh ET (2, 6). Conversely, recent studies focusing on specific subpopulations, such as women with fewer oocytes retrieved, indicated that the stage of the embryo at ET did not predict live birth rates per transfer or the CLBR (7). These findings suggested that it may be premature to universally endorse the advantages of blastocyst-stage fresh ET for all women undergoing IVF-ET. Women with particular characteristics might still achieve similar or better outcomes with cleavage-stage fresh ET.

Endometrial thickness (EMT) is commonly utilized as a marker to assess endometrial receptivity and is routinely assessed during controlled ovarian stimulation (COS) in IVF-ET (8). An EMT less than 7 or 8 mm, measured on the day of the human chorionic gonadotropin (hCG) trigger, is classified as thin endometrium (TE) (9). Previous research has identified specific cutoff values for EMT that correlate with declining live birth rates (10, 11). Interestingly, the dividing cutoff value varied for women receiving blastocyst-stage fresh ET and those receiving cleavage-stage fresh ET, indicating a possible interaction between the embryo stage at fresh ET and EMT on the CLBR. However, this aspect has not been extensively explored (10). To our knowledge, only one study has examined the impact of the embryo stage at frozen-thawed embryo transfer (FET) on LBR and CPR in TE women. The results showed a significant superiority in the blastocyst group over the cleavage group (12). The limitation was that the study failed to assess fresh ET outcomes, as well as cumulative outcomes. As a result, little is known about the optimal embryo stage at fresh ET for women with TE.

The primary drawback of the blastocyst-stage fresh ET approach is the potential loss of embryos with lower developmental prospects during the self-selection process, resulting in fewer embryos being transferred and preserved. In contrast, cleavage-stage fresh ET typically involves a greater number of embryos to be transferred (13). In women with TE, impaired endometrial receptivity is the leading cause of embryo implantation failure (14). In this population, which is of greater importance: the quality or the quantity of embryos transferred? High-quality studies are required to answer the question. The study aims to compare the effect of blastocyst-stage fresh ET and cleavage-stage fresh ET on the CLBR in TE women, as well as the pregnancy and neonatal outcomes after the single fresh embryo transfer. In addition to the original cohort, we also established a matched cohort with comparable baseline characteristics and embryo culture outcomes, to facilitate a robust analysis.

## 2 Materials and methods

### 2.1 Ethical approval

The study was approved by the Ethics Committee of the Affiliated Hospital of Shandong University of Traditional Chinese Medicine (2024-061-01-KY). The database utilized for analysis was fully anonymized. No personally identifiable information was collected or used. Informed consent was obtained from all patients before receiving assisted reproductive technology (ART) treatment.

### 2.2 Study design and population

This is a three-center retrospective cohort study, including TE women scheduled for their first IVF-ET cycle between September 2017 and January 2022 at the following centers: (1) The Affiliated Hospital of Shandong University of Traditional Chinese Medicine; (2) The Second Affiliated Hospital of Shandong University of Traditional Chinese Medicine; and (3) Shanxi Maternal and Child Health Care Hospital. All included women were followed up for at least 2 years. TE was defined as an EMT <= 8 mm, measured in the mid-sagittal plane by transvaginal ultrasound on the day of hCG administration, consistent with previous studies (15, 16).

The included population was divided according to the embryo stage at the time of fresh ET: cleavage-stage embryo (day 3) and blastocyst-stage embryo (day 5). To mitigate the influence of variable proportions of vitrified embryos between groups, surplus embryos from all participants were vitrified on day 5 post-oocyte retrieval. Decisions regarding the timing of fresh ET and the developmental stage of the embryos to be transferred were collaboratively made by the patients and their clinicians. Women who met the following criteria were excluded: (1) Women had undergone preimplantation genetic testing or diagnosis cycles; (2) Women had undergone cycles with cryopreserved oocytes and/or donor oocytes; (3) Women who had uterine abnormalities, other than TE; (4) Women who had no oocytes retrieved, no available zygotes observed on D1 and no available embryos observed on day 3. Additionally, to account for the preference of clinicians to perform blastocyst-stage fresh ET in cases with a higher number of embryos available on day 3, propensity score matching (PSM) was employed to adjust for potential confounding factors in baseline characteristics and embryonic development outcomes.

### 2.3 Ovarian stimulation protocol

The gonadotrophin-releasing hormone agonist (GnRH-a) protocol and GnRH antagonist (GnRH-ant) protocol were applied for pituitary downregulation in this study and were tailored to individual characteristics and clinician preferences.

For the luteal-phase GnRH-a protocol, triptorelin acetate (0.1 mg; Ferring Pharmaceuticals, Switzerland) was administered to the patients once daily for 14 days starting from the mid-luteal phase. Gonadotropin (Gn) administration commenced upon confirmation of complete pituitary desensitization, indicated by FSH < 5 mIU/mL, LH < 5 mIU/mL, E2 < 50 pg/mL, and EMT < 5 mm. For the ultralong GnRH-a protocol, patients were administered an intramuscular injection of long-acting leuprorelin Acetate (3.75 mg, Livzon Pharmaceutical, China) between days 1 and 3 of the menstrual cycle. Ultrasound scans and sex hormone examinations were performed 28 days later. Ovarian stimulation was started when complete pituitary desensitization was confirmed as previously described. The starting dosage of recombinant follicle-stimulating hormone (r-FSH) (Gonal F, Merck Serono S.p.A, Modugno, Italy) was 150–300 IU daily. The starting dosage of Gn was determined based on individual patient baseline characteristics. The follow-up dosage was adjusted according to the patient’s ovarian response. For the GnRH-ant protocol, similar criteria for starting dose and dosage adjustments were applied, with a daily 0.25 mg dose of cetrorelix acetate (Cetrotide; Merck Serono, Germany) starting from day 6 of stimulation, or when the leading follicle reached 12-14 mm in diameter, continuing until the ovulation trigger day. When two or more follicles measured 18 mm or more, a 250 μg dose of recombinant hCG (Ovidrel, Merck Serono S.p.A., Modugno, Italy) was administrated to trigger ovulation. Oocyte retrieval was performed 34–36 h after hCG injection.

### 2.4 Laboratory procedures and fresh embryo transfer

The fertilization method for the retrieved oocytes was determined based on the quality of the partner’s semen. In general, intracytoplasmic sperm injection (ICSI) was indicated in case the total progressive motile sperm count was <5×10^6^, the normal morphology of the sperm was <1%, or there was a history of complete fertilization failure in a previous IVF cycle. The embryos were cultured individually in sequential G-series media (Vitrolife, Göteborg, Sweden). The incubation conditions were set at 6% CO2, 5% O2, and 37.0°C (C200 CO_2_ Incubator, Labotect Labor-Technik-Göttingen GmbH, Göttingen, Germany). Fertilization was assessed 16-18 hours after insemination and embryo development was evaluated daily until the day of ET. The quality of the embryos on day 3 was assessed 67-69 hours after insemination, based on the blastomere count, regularity, embryonic fragmentation rate, and the presence of multinucleation. Good quality embryos (GQE) at the cleavage-stage were determined when the blastomere count ranged from 7-14, the sizes were uniform, no multinucleation was observed, and the fragmentation rate was < 25% (17). In the cleavage group, up to 2 cleavage-stage GQEs were chosen for transfer on day 3. In the blastocyst group, all embryos were extended-cultured until day 5 to reach the blastocyst-stage. These embryos were then evaluated according to the Gardner-Schoolcraft scoring system and the expert consensus in China (17, 18). Blastocyst-stage embryos scoring ≥ 3BC or 3CB were classified as transferable, and those scoring ≥ 3BB were classified as blastocyst-stage GQEs. One to two blastocyst-stage GQEs were transferred on day 5.

### 2.5 Vitrification and frozen-thawed embryo transfer

For both groups, embryos not utilized in fresh ET were cultured to the blastocyst stage. Those achieving a grade of 3BC or higher were cryopreserved using a closed vitrification system (CBS-ViT-HS, CryoBioSystem, L’Aigle, France) with high-security straws. Dimethyl sulfoxide, ethylene glycol, and sucrose were used as cryoprotectants (Irvine Scientific Freeze Kit, Irvine Scientific, Newtown Mount Kennedy, Ireland). For ovulatory women, the natural cycle protocol is recommended for FET. Otherwise, a hormone replacement cycle is recommended. A maximum of two blastocyst-stage embryos were transferred per woman in FET cycles.

### 2.6 Outcome measurement

The primary outcome was the CLBR per woman, with the number of transfer cycles as a time component. Live birth was defined as the delivery of at least one viable child at 24 weeks or later, according to the Reference-International Committee for Monitoring Assisted Reproductive Technology (ICMART) and the World Health Organization (WHO) (19). The CLBR was calculated as the proportion of women who achieved at least one live birth, either from the initial fresh ET cycle or subsequent FET cycles within at least 24 months of follow-up. Only the first live birth was counted in the CLBR. A positive hCG test was defined as a serum hCG level >= 25mIU/ml, measured on the 14 days after ET; Clinical pregnancy was identified by the presence of one or more intrauterine gestational sacs on ultrasound; Implantation rate (IR) was defined as the ratio of the number of gestational sacs to the total number of transferred embryos. Neonatal outcomes were also assessed, including gestational age, birth weight, rates of preterm birth (PTM, <37 weeks of gestation), low birth weight (LBW, <2.5 kg at birth), high birth weight (HBW, >4 kg at birth), small for gestational age (SGA), and large for gestational age (LGA). We defined SGA and LGA as birth weights < 10th or > 90th percentile for gestational age of the average body weight stratified by gestational age and neonatal sex, according to the current official Chinese standards (20).

### 2.7 Statistical analysis

The standard PSM method was conducted, using nearest neighbor matching with a caliper of 0.02 in a 1:1 ratio to estimate the propensity score via logistic regression, adjusting for key predictors for IVF success as previously described (21, 22), as follows: age of women at enrollment, body mass index (BMI), duration of infertility, type of infertility, infertility indicators, antral follicle count (AFC), COS protocol, insemination method, number of oocytes retrieved, number of two pronucleus (2PN) zygotes, and number of GQEs on day 3. Propensity scores before and after PSM were visualized using density plots to test the balance. Baseline characteristics before fresh ET were assessed for normality using the Shapiro-Wilk test and were expressed as the median and interquartile range (IQR, 25th–75th) for continuous variables, and as frequency (n) and percentage (%) for categorical variables. Group differences were analyzed using the Mann-Whitney test for continuous variables and the chi-square test for categorical variables. The standardized mean difference (SMD) was used to examine the balance of baseline variables before fresh ET between groups, with a meaningful imbalance set at a value > 0.1.

Pregnancy and neonatal outcomes after the fresh embryo transfer were compared between groups in the matched cohort. We also calculated the hazard ratio (HR) by applying the Kaplan-Meier curve analysis to visualize the CLBR for the cleavage and blastocyst groups, taking the number of transfer cycles as a time component. Patients who did not deliver a live birth until the end of follow-up or used up all embryos with no live birth were censored. The log-rank test was used to measure the difference in Kaplan-Meier curves between groups. To further reduce the bias caused by confounding factors, multivariate Cox proportional hazards models were applied to test the impact of embryo stage at fresh ET on the CLBR before and after PSM. We employed several statistical methods to ensure the robustness of the Cox models: (1) The Schoenfeld residual test was applied to test the proportional hazards assumption. (2) Restricted cubic spline methods were applied to test the linearity of continuous variables such as age and BMI, against the log-transformed HR. (3) Correlations between variables were tested. For those variables that were highly correlated, we eliminated one from the model and retained the other.

In our sensitivity analysis, we addressed the significant proportion of women who exhausted all available embryos without achieving a live birth. Defining this group of women as merely censored could lead to an overestimation of the CLBR. As a result, we additionally established multivariate Fine-Gray competing risk models, defining the use of all embryos with no live birth as a competing event for cumulative live birth. The cumulative incidence function (CIF) curve was also drawn to visualize the comparison between groups. Finally, we repeated the above multivariate regression analysis for people with an EMT < 7 mm to test the robustness of the results under stricter TE diagnostic criteria. Differences were considered statistically significant when the two-sided p-value was < 0.05. All univariable and multivariable analyses above to calculate HRs were performed in the original cohort and the matched cohort, before and after PSM.

## 3 Results

**Figure 1:**
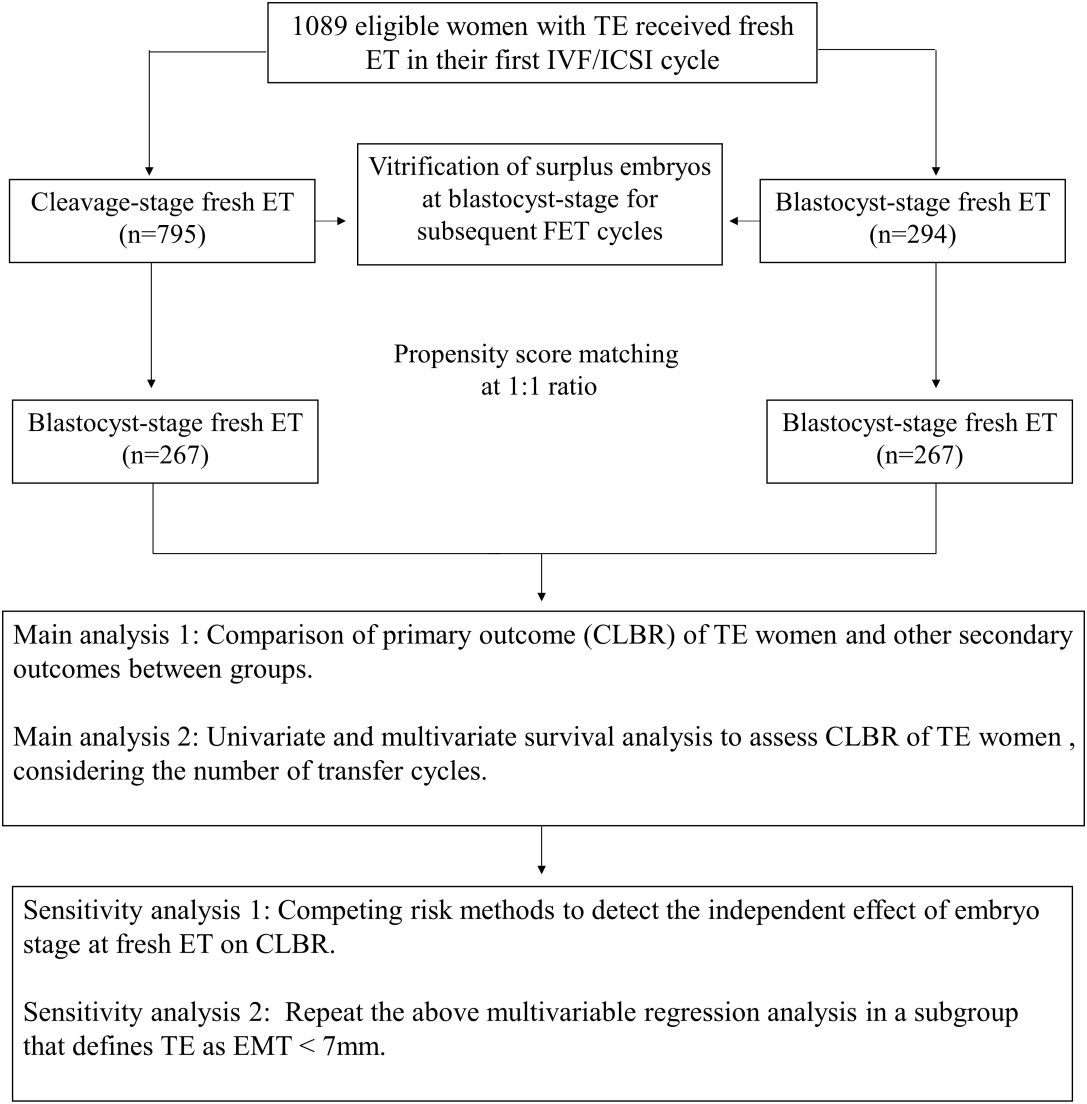
Flow chart of the study. TE, Thin endometrium; ET, Embryo transfer; IVF/ICSI, *In vitro* fertilization/Intracytoplasmic sperm injection; CLBR, Cumulative live birth rate; EMT, Endometrium thickness.

**Figure 2:**
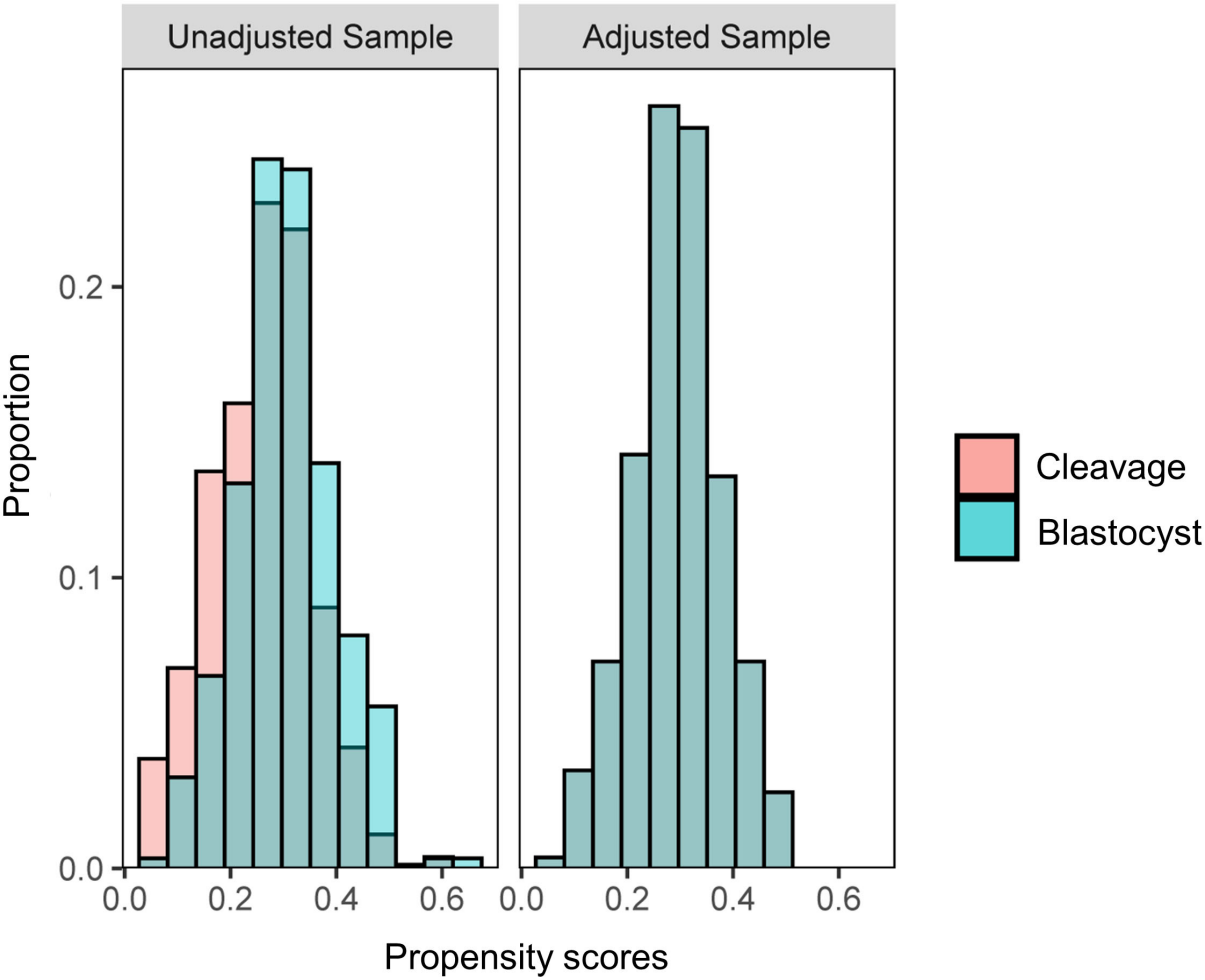
A histogram of density demonstrating the propensity score distribution between groups before and after propensity score matching to test balance.

A total of 2992 women were screened, out of which 1270 women received fresh ET. After eligibility assessment, 1089 women were included for analysis, of which 795 women received cleavage-stage fresh ET on day 3 after oocyte retrieval, and 294 women received blastocyst-stage fresh ET on day 5 after retrieval. After PSM, 267 women in each group were matched (**Figure 1**). A density histogram was generated to visually compare PS before and after matching (**Figure 2**). In the matched cohort, the PS for the cleavage group and blastocyst group showed a very similar distribution.

### 3.1 Baseline and stimulation cycle characteristics

The baseline and stimulation cycle characteristics before and after PSM are shown in **Table 1**. In the original cohort, the duration of infertility was significantly longer in the cleavage group (median days were 3 for both groups, IQR of 1-5 and 1-4, SMD=0.292). More women in the cleavage group had never been pregnant before (29.9% versus 24.7%, SMD=0.118). Significant differences in infertility indicators were noted between the groups (SMD=0.327). A larger proportion of women received the GnRH agonist protocol for COS and ICSI for fertilization in the cleavage group than in the blastocyst group (77.5% versus 70.1%, SMD=0.169; 21.4% versus 15.6%, SMD=0.148). The days of COS were significantly longer, and the total dose of Gn (mIU/ml) was significantly higher in the cleavage group (median days were 10 and 9, IQR of 8-11 for both groups, SMD=0.132; median dose was 1800 for both groups, IQR of 1350-2475 and 1350-2325, respectively, SMD=0.117). The serum progesterone level (ng/ml) on the day of hCG trigger was higher in the cleavage group, compared to the blastocyst group (median levels of 0.80 and 0.73, IQR of 0.57-1.02 and 0.51-0.96, SMD=0.169). As expected, women scheduled for blastocyst fresh ET generally had more GQEs on day 3 (median of 2 and 3, IQR of 1-4 and 1-5, respectively, SMD=0.130).

**Table 1:** Baseline and stimulation cycle characteristics in the original cohort and matched cohort after propensity score matching.

Characteristic	Original cohort			After propensity score matching		
	Cleavage (n=795)	Blastocyst (n=294)	SMD	Cleavage (n=267)	Blastocyst (n=267)	SMD
Age (years)	34.00 [30.00, 39.00]	34.00 [30.00, 38.00]	0.026	34.00 [29.00, 39.00]	34.00 [30.00, 38.00]	0.004
BMI (kg/m2)	23.44 [21.30, 26.04]	23.40 [21.16, 25.76]	0.016	23.44 [21.09, 25.48]	23.35 [21.08, 25.64]	0.003
Duration of infertility	3.00 [1.00, 5.00]	3.00 [1.00, 4.00]	0.292	2.00 [1.00, 4.00]	2.50 [1.50, 4.00]	0.047
Type of infertility			0.118			0.026
Primary infertility	235 (29.9%)	72 (24.7%)		70 (26.2%)	67 (25.1%)	
Secondary infertility	551 (70.1%)	220 (75.3%)		197 (73.8%)	200 (74.9%)	
Infertility indicators			0.327			0.092
Male factor	62 (7.8%)	21(7.1%)		19 (7.1%)	19 (7.1%)	
Tubal factor Unexplained	519 (65.3%)	219 (74.5%)		204 (76.4%)	200 (74.9%)	
	87 (10.9%)	18 (6.1%)		17 (6.4%)	18 (6.7%)	
Endometriosis	25 (3.1%)	16 (5.4%)		9 (3.4%)	10 (3.7%)	
DOR	35 (4.4%)	3 (1.0%)		1 (0.4%)	3 (1.1%)	
Anovulatory	67 (8.4%)	17 (5.8%)		17 (6.4%)	17 (6.4%)	
Antral follicle count	11.00 [7.00, 15.00]	10.00 [7.00, 15.00]	0.099	10.00 [7.00, 14.00]	10.00 [7.00, 14.00]	0.011
COS protocol			0.169			0.017
Agonist	661 (77.5%)	206 (70.1%)		195 (73.0%)	193 (72.3%)	
Antagonist	179 (22.5)	88 (29.9%)		72 (27.0)	74 (27.7%)	
Total gonadotrophin (IU/L)	1800 [1350, 2475]	1800 [1350.0, 2325.0]	0.117	1800 [1350, 2362]	1800 [1350,2325]	0.098
Days of COS	10 [8.00, 11.00]	9 [8.00, 11.00]	0.132	10.00 [8.00, 11.00]	9.00 [8.00, 11.00]	0.160
Endometrial thickness on day of hCG trigger (mm)	7 [6.5, 7.5]	7 [6.5, 7.5]	0.029	7.0 [6.5, 7.5]	7.0 [6.5, 7.5]	0.096
Estradiol on day of hCG trigger (pg/ml)	2133 [1361, 3000]	2205 [1415, 3000]	0.013	2230 [1489, 3000]	2197 [1374, 3000]	0.015
Progesterone on day of hCG trigger (ng/ml)	0.80 [0.57, 1.02]	0.73 [0.51, 0.96]	0.169	0.80 [0.59, 1.03]	0.74 [0.52, 0.98]	0.166
No. of oocytes retrieved	7.00 [4.00, 10.00]	7.00 [4.00, 10.75]	0.041	7.00 [4.00, 11.00]	7.00 [4.00, 10.00]	0.112
Insemination method			0.148			0.031
IVF	625 (78.6%)	248 (84.4%)		228 (85.4%)	225 (84.3%)	
ICSI	170 (21.4%)	46 (15.6%)		39 (14.6%)	42 (15.7%)	
No. of 2PN zygotes	4.00 [2.00, 7.00]	4.00 [2.00, 7.00]	0.057	4.00 [2.00, 7.00]	4.00 [2.00, 7.00]	0.020
No. of GQE on day 3	2.00 [1.00, 4.00]	3.00 [1.00, 5.00]	0.130	3.00 [1.00, 5.00]	3.00 [1.00, 5.00]	0.012

Continuous data are represented as median (25th and 75th percentile) because of nonnormal distribution, and categorical variables are represented as number (%). SMD, Standardized mean difference (given as absolute value); BMI, Body mass index; DOR, Diminished ovarian reserve; COS, Controlled ovarian stimulation; hCG, Human chronic gonadotrophin; IVF, *In-vitro* fertilization; ICSI, Intracytoplasmic sperm injection; 2PN, Two pronucleus; GQE, Good-quality embryos.

All variables of imbalance mentioned previously were adjusted by PSM. No differences in variables were observed after PSM, except for days of COS, serum progesterone level on the day of hCG trigger, and number of oocytes retrieved, all of which were further adjusted for in subsequent multivariable analysis.

### 3.2 Pregnancy and neonatal outcomes after fresh embryo transfers

**Table 2** presents the pregnancy and neonatal outcomes for fresh embryo transfers within the matched cohort. In this cohort, 476 embryos were transferred to women receiving cleavage-stage fresh ET, and 269 embryos were transferred to women receiving blastocyst-stage fresh ET. A larger proportion of women in the cleavage group (78.3%) received double embryo transfer (DET), compared to almost all women in the blastocyst group (99.3%) receiving single embryo transfer (SET) (p < 0.001). The positive hCG test rate, CPR, IR, and LBR were all comparable between the groups (all p values > 0.05). In the cleavage group, multiple pregnancies accounted for 36.9% of all clinical pregnancies, while twin live births accounted for 26.0% of live births. Correspondingly, no multiple pregnancies occurred in the blastocyst group. Regarding neonatal outcomes, the proportion of cesarean sections was similar (72.7% versus 73.1%, p=0.894). No significant difference was observed in terms of gestational age or preterm birth rate (all p values > 0.05). However, the median birth weight (kg) was significantly higher in the blastocyst group (median weights of 2.95 and 3.40, IQR of 2.55-3.30 and 3.20-3.60, respectively, p < 0.001). Furthermore, the LBW rate was significantly higher in the cleavage group (23.71% versus 5.97%, p=0.005). After taking gestational age and neonatal sex into consideration, women who received cleavage-stage fresh ET also had a higher incidence of SGA, compared to those receiving blastocyst-stage fresh ET (20.62% versus 7.46%, p=0.037). The rates of HBW and LGA were comparable between the groups.

**Table 2:** Pregnancy and neonatal outcomes of fresh embryo transfers in the matched cohort.

Characteristic	Cleavage (n=267)	Blastocyst (n=267)	P value
Fresh ET cycle outcomes			
No. of embryos transferred	476	269	
Ratio no. of embryos transferred per cycle (%)			< 0.001^a^
One	58 (21.7%)	265 (99.3%)	
Two	209 (78.3%)	2 (0.7%)	
Positive hCG test rate	125 (46.8%)	110 (41.2%)	0.222
Clinical pregnancy rate	103 (38.6%)	93 (34.8%)	0.419
Multiple pregnancy rate per pregnancy	38 (36.9%)	0	-
Implantation rate (per embryo)	141 (29.6%)	93 (34.6%)	0.188
Live birth rate	77 (28.8%)	67 (25.1%)	0.380
Twin live birth rate per live birth	20 (26.0%)	0	-
Cesarean section ratio	56 (72.7%)	49 (73.1%)	0.894
Gestational age (weeks)	38.29 [37.29, 39.57]	39.14 [38.00, 39.71]	0.076
Preterm birth rate (< 37 weeks)	15 (19.48%)	6 (8.96%)	0.122
Birth weight (kg)	2.95 [2.55, 3.30]	3.40 [3.20, 3.60]	< 0.001^a^
Low birth weight rate (< 2.5kg)	23 (23.71%)	4 (5.97%)	0.005^a^
High birth weight rate (> 4kg)	3 (3.09%)	6 (8.96%)	0.203
SGA rate	20 (20.62%)	5 (7.46%)	0.037^a^
LGA rate	10 (10.31%)	15 (22.39)	0.058

^a^A p-value < 0.05 indicates that the difference is statistically significant. ET, embryo transfer; hCG, Human chronic gonadotrophin; FET, Frozen-thawed embryo transfer; HRT, Hormone replacement therapy; GnRH-a, Gonadotrophin-releasing hormone agonist; SGA, Small for gestational age; LGA, Large for gestational age; IVF-ET. Continuous data are represented as median (25th and 75th percentile) because of nonnormal distribution, and categorical variables are represented as number (%).

### 3.3 Cumulative outcomes

**Figure 3:**
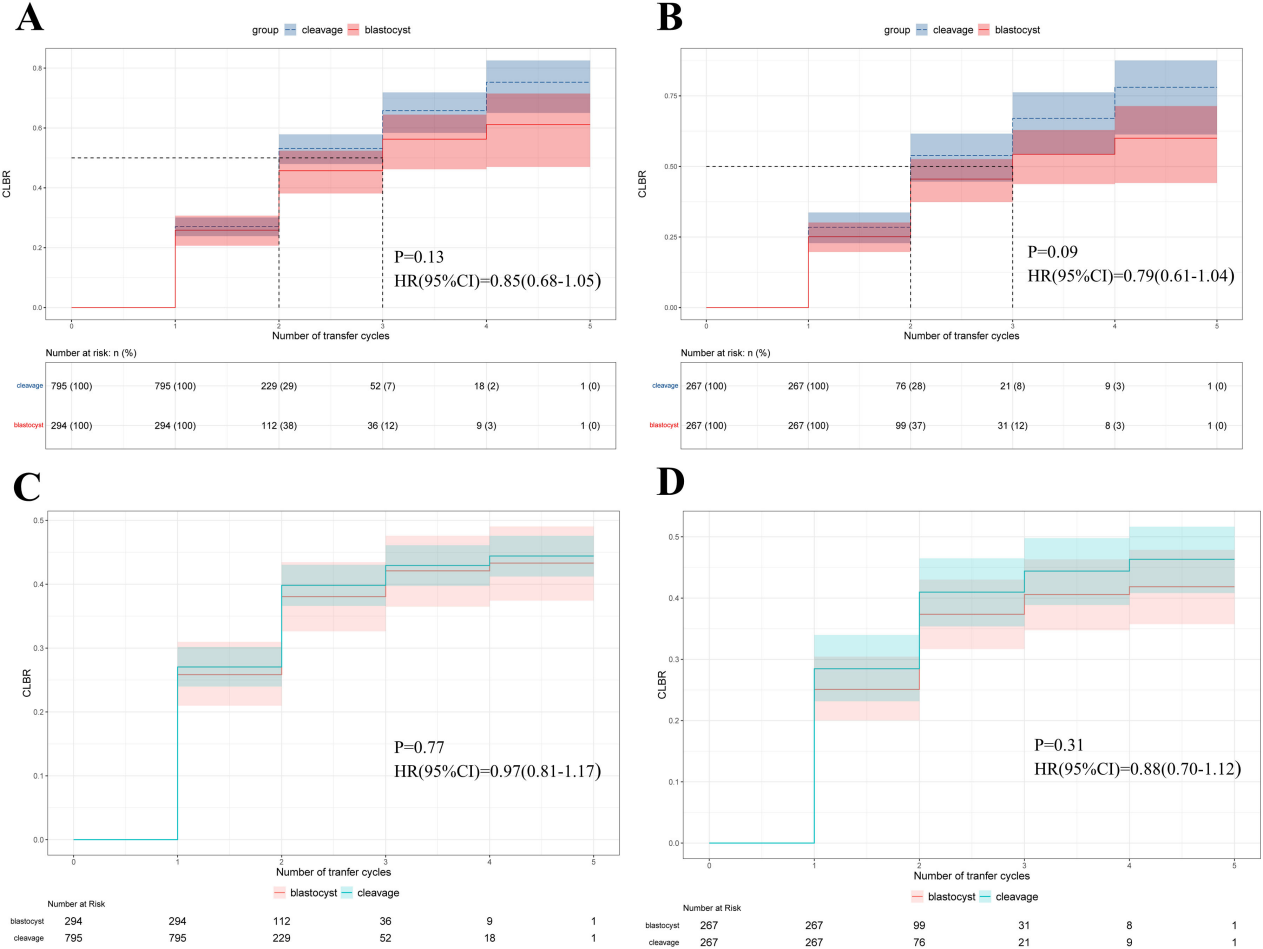
Survival analysis of the cumulative live birth rate (CLBR) (y axis) as the event of interest and number of embryo transfer cycles (x axis) as the time component. **(A)** Kaplan-Meier curves for the original cohort. **(B)** Kaplan-Meier curves for the matched-controlled cohort. **(C)** Cumulative incidence function curves for the original cohort. **(D)** Cumulative incidence function curves for the matched-controlled cohort.

No significant difference was observed in the CLBR, between women receiving cleavage-stage and blastocyst-stage fresh ET (41.9% versus 37.5%, p=0.33, **Table 3**). At the end of follow-up, 28 women in the cleavage group and 43 women in the blastocyst group remained embryos with no live births achieved (10.5% versus 16.1%, respectively, p=0.07). The Kaplan-Meier analysis of the matched cohort revealed that the CLBR after 5 times of embryo transfers was 78.1% in the cleavage group and 60.0% in the blastocyst group (p=0.09, HR=0.79, 95% CI= 0.61-1.04, **Figure 3B**). Similar results were shown in the cohort before PSM, where the CLBR after 5 times of embryo transfers was 76.3% in the cleavage group and 61.2% in the blastocyst group (p=0.13, HR=0.85, 95% confidence interval (CI)= 0.68-1.05, **Figure 3A**).

**Table 3:** The cumulative pregnancy outcomes of IVF-ET cycles in the matched cohort.

Characteristic	Cleavage (n=267)	Blastocyst (n=267)	P value
Total no. of ET cycles (Fresh and FET)	384	417	
Total no. of embryo transferred	600	430	
CLBR	112 (41.9%)	100 (37.5%)	0.331
Patients remained embryos until the end of follow-up	28 (10.5%)	43 (16.1%)	0.074

IVF-ET, *In vitro* fertilization and embryo transfer; FET, Frozen-thawed embryo transfer; CLBR, Cumulative live birth rate. Continuous data are represented as median (25th and 75th percentile) because of nonnormal distribution, and categorical variables are represented as number (%).

After performing the statistical diagnostics, multivariable Cox proportional hazard models were established to assess the effect of embryo stage at fresh ET on the CLBR, adjusting for age, BMI, type of infertility, duration of infertility, infertility indicators, AFC, COS protocol, days of COS, EMT on the day of hCG trigger, serum progesterone level on the day of hCG trigger, number of oocytes retrieved, insemination method and number of GQEs on day 3 after oocyte retrieval. In the matched cohort, the CLBR was not significantly associated with the embryo stage at fresh ET (p=0.14, HR=0.80, 95% CI=0.60-1.07, **Table 4**). The results were consistent for the original cohort before PSM (p=0.30, HR=0.88, 95% CI=0.70-1.12, **Table 4**).

**Table 4:** Hazard ratio calculated by Cox proportional hazard models and Fine-grey competing risk models on CLBR in women with thin endometrium.

Original cohort				After propensity score matching	
Selected populations and multivariable models	Covariate strata	p value	Hazard ratio (95%CI)	p value	Hazard ratio (95%CI)
(A) TE defined as <= 8 mm					
Cox proportional hazard models ^a^					
Stage of embryo at fresh ET	Cleavage-stage	Reference	1	Reference	1
	Blastocyst-stage	0.30	0.88(0.70-1.12)	0.14	0.80(0.60-1.07)
Fine-gray competing risk models ^b^					
Stage of embryo at fresh ET	Cleavage-stage	Reference	1	Reference	1
	Blastocyst-stage	0.59	0.95(0.78-1.15)	0.41	0.91(0.71-1.15)
(B) TE defined as < 7 mm					
Cox proportional hazard models ^a^					
Stage of embryo at fresh ET	Cleavage-stage	Reference	1	Reference	1
	Blastocyst-stage	0.14	0.72(0.46-1.12)	0.78	0.91(0.49-1.72)
Fine-gray competing risk models ^b^					
Stage of embryo at fresh ET	Cleavage-stage	Reference	1	Reference	1
	Blastocyst-stage	0.18	0.77(0.53-1.12)	0.98	1.01(0.58-1.74)

CI, Confidence interval; TE, Thin endometrium; ET, Embryo transfer.

^a^Adjusted for age, BMI, type of infertility, duration of infertility, infertility indicators, AFC, COS protocol, days of COS, EMT on the day of hCG trigger, serum progesterone level on the day of hCG trigger, number of oocytes retrieved, insemination method and number of GQE on day 3 after oocyte retrieval.

^b^Defining using up all embryos while achieving no live birth as a competing event to cumulative live birth. Adjusted covariables included age, BMI, type of infertility, duration of infertility, infertility indicators, AFC, COS protocol, days of COS, EMT on the day of hCG trigger, serum progesterone level on the day of hCG trigger, number of oocytes retrieved, insemination method and number of GQE on day 3 after oocyte retrieval.

### 3.4 Sensitivity analysis

Initially, after excluding competing risk events for the CLBR, the CIF curve was plotted, demonstrating a trend similar to that of the Kaplan-Meier curves in the original cohort (p=0.77, HR=0.97, 95% CI= 0.81-1.17, **Figure 3C**), and the cohort after PSM (p=0.31, HR=0.88, 95% CI= 0.70-1.12, **Figure 3D**). We also established multivariate Fine-Gray competing risk models to validate the robustness of the Cox models, and the results remained consistent in the original cohort (p=0.59, HR=0.95, 95% CI=0.78-1.15, **Table 4**), and the cohort after PSM (p=0.41, HR=0.91, 95% CI=0.71-1.15, **Table 4**). Moreover, all multivariable analyses were repeated in a cohort in which TE was defined as an EMT < 7 mm, and the results remained robust (**Table 4**).

## 4 Discussion

To our knowledge, this is the first study to focus exclusively on women with TE, indicating that the embryo stage at fresh ET does not affect the CLBR for TE women.

TE, with an approximate incidence of 1%-2.5% (23), depending on whether the cutoff is set at 7 or 8 mm, is often regarded as a principal indicator of impaired endometrial receptivity, resulting in unfavorable outcomes in IVF-ET (24). Historically, it was recommended to cancel the fresh ET in cases of TE during IVF-ET cycles. However, recent studies have challenged this approach, arguing that TE should no longer serve as a basis for canceling fresh ET at any EMT value (25–27). In this case, if fresh ET with TE is permitted, which embryo stage is optimal? Our results answered the question by revealing a comparable CLBR between the cleavage group and the blastocyst group. Moreover, the Kaplan-Meier curve showed that the median number of embryo transfer cycles was 2 and 3 in the cleavage group and the blastocyst group, respectively. Cleavage-stage fresh ET tended to be advantageous over blastocyst-stage fresh ET, although the observed difference was not statistically significant. The number of transferred embryos was much higher in the cleavage group, predominantly as double embryo transfers (DET). The advantage in embryo numbers was specific to the fresh ET cycle, as all embryos were vitrified at the blastocyst-stage for both groups, regardless of the stage of fresh ET. Our finding contrasts with the study by Zhang et al., which reported that women receiving blastocyst-stage embryos in FET cycles achieved higher LBR, compared to those receiving cleavage-stage embryos (12). This discrepancy can be attributed to differences in study aims, populations, and primary outcomes.

As for studies focusing on the embryo stage at fresh ET in the unselected population, a national population-based retrospective cohort study by Cameron et al. showed that blastocyst-stage fresh ET was associated with higher CLBR (6). However, this study’s limitation was the unavailability of data on the embryo stages transferred in FET cycles, which could affect the results. The primary distinction between our study and that of De Vos A. was their implementation of a strict SET policy at any embryo stage, leading to an absolute advantage of blastocyst-stage embryos at fresh ET. In terms of the CLBR, they observed no significant difference between groups, which was in line with our results (5). De Croo I et al. reported that women receiving blastocyst-stage fresh ET achieved high CLBR compared to those receiving cleavage-stage fresh ET (28), but the results were limited by the use of different embryo-freezing protocols, as the vitrification protocol has been proven to be superior to the slow freezing protocol (29). An updated Cochrane systematic review demonstrated that blastocyst-stage fresh ET is associated with higher CPR and LBR than cleavage-stage fresh ET, although the evidence was rated from low to moderate based on 32 previous randomized controlled trials (RCTs). Interestingly, the cumulative CPR did not differ between groups (30). At the end of the review, the authors called for more studies to report cumulative, long-term pregnancy outcomes such as the CLBR, and studies focusing on specific subpopulations. The review also reported comparable multiple CPR between groups and a higher cycle cancellation rate in the blastocyst group. In our study, multiple pregnancies accounted for 36.9% of pregnancies after fresh ET in the cleavage group, highlighting a critical limitation of the cleavage-stage fresh ET approach (31, 32).

To explain our finding of the unexpectedly positive impact of cleavage-stage embryos on the CLBR in TE women, which is contrary to prevailing beliefs in the general population, we hypothesize that it is because, for TE women, the implantation rate of a single embryo is declining, regardless of the embryo stage. In TE women, impaired endometrial receptivity plays a preeminent role, while the influence of embryo quality relatively diminishes. Additionally, the sheer number of embryos available for transfer in the cleavage stage may confer an advantage, contributing to our findings.

Since there is still insufficient evidence to determine which embryo stage is optimal for women receiving fresh ET, one study by Cornelisse S analyzed preferences regarding the timing of embryo transfer among 445 women (33). The results showed that patients highly value effectiveness in terms of the CLBR and the number of opportunities (number of embryos available for transfer), regardless of the number of transfers needed until pregnancy and the impact on quality of life. According to our study results, women with TE may prefer cleavage-stage fresh ET because there are more transfer opportunities, with the same CLBR as blastocyst-stage embryo transfer. Clinicians should take this into account before making the choice.

To assess the potential effects of the embryo stage at fresh ET on neonatal outcomes after live birth, we conducted an exploratory analysis of women with live births. According to our results, cleavage-stage fresh ET was associated with increased rates of LBW and SGA. We propose that these higher incidences in the cleavage-stage group may primarily result from multiple pregnancies, which are more common due to the higher frequency of DET (34). Additionally, extended culture periods may contribute to higher birth weights, as suggested by prior research (35). LBW and SGA are considered the main causes of neonatal mortality and multiple long-term adverse health conditions (36). Previous publications by Zheng et al. and Guo et al. have indicated that the offspring of TE women are inherently at an elevated risk of LBW and SGA and our study confirms that cleavage-stage fresh ET may exacerbate this risk (37, 38). Consequently, it is recommended that TE women who receive cleavage-stage fresh ET should be provided with additional prenatal care, such as nutritional supplements, to mitigate the risk of delivering an LBW or SGA fetus.

There were several strengths in our study. First, in line with the recommendations from the 23rd annual report of ESHRE, we selected the CLBR of one IVF cycle as the primary outcome because it included the fresh ET cycle and all subsequent FET cycles, thereby more accurately reflecting real-world scenarios (3). Each patient was followed up for at least 2 years, ensuring that most patients used all embryos obtained from one IVF cycle (whether live births or not). Second, we derived a comparable cohort from the original cohort of real-world practice using PSM to adjust for potential confounding factors, whether from baseline characteristic imbalances or subjective clinical decisions (39). A variety of analyses were also conducted to ensure the robustness of the findings. Furthermore, we intentionally excluded those patients with other intracavitary diseases in addition to TE, such as intracavitary lesions, hydrosalpinges, and Asherman syndrome, to avoid confounding variables that could significantly impact the outcome assessments within the TE group—a notable limitation in previous TE studies (40).

Our study was limited by its retrospective design and the inconsistency in the categories of data collected across participating centers, especially the endometrial pattern, which is considered another dependent variable of endometrial receptivity (16). In addition, although clinical protocols and embryo culture conditions were theoretically standardized across centers based on Chinese guidelines and expert consensus, discrepancies in the practices of clinical and laboratory personnel may still exist.

For the interpretation of our findings, it is not possible to conclude that a clear advantage exists for one of cleavage-stage fresh ET or blastocyst-stage fresh ET for patients with TE, and furthermore, the ethically relevant risks triggered by multiple pregnancies accompanied by cleavage-stage embryos need to be emphasized. According to the American College of Obstetricians and Gynecologists’ Practice Bulletin, multiple pregnancies are not only associated with increased risks of neonatal death and long-term complications but are also economically linked to significantly higher costs (41). Therefore, clinicians have a duty to inform patients who are pursuing single transfer pregnancy rates and opting for the transfer of two cleavage-stage embryos of the potential long-term risks associated with this choice, which may be selectively overlooked by patients. Additionally, we recommend that clinicians introduce the concept of cumulative live birth rates to patients, which may help them view the benefits of IVF treatment from a more objective perspective, thereby avoiding potential future disputes.

In conclusion, the present study indicated that for TE women there is no optimal choice between cleavage-stage fresh ET or blastocyst-stage fresh ET, in terms of CLBR. However, receiving cleavage-stage fresh ET may increase the risk of LBW and SGA in offspring. In the clinical practice involving TE women, clinicians should consider multiple factors, including neonatal risks, the patient preference, history of intracavitary diseases, the risk of multiple pregnancies, and the possibility of cycle cancellation, when deciding whether to extend culture to the blastocyst-stage.

## Data Availability

The raw data supporting the conclusions of this article will be made available by the authors, without undue reservation.
